# A Prospective Study Examining Audiometry Outcomes Following Teprotumumab Treatment for Thyroid Eye Disease

**DOI:** 10.1089/thy.2023.0466

**Published:** 2024-01-16

**Authors:** Raymond S. Douglas, Emanuil Parunakian, Joseph Tolentino, Emil Malkhasyan, June Geng, Michele Sherman, Shoaib Ugradar

**Affiliations:** ^1^ Department of Orbital and Oculoplastic Surgery, Private Practice, Beverly Hills, California, USA.; ^2^ Department of Audiology, The Towers, Audiology Center, Cedars Sinai Medical Center, Los Angeles, California, USA.

**Dear Editor:**

Teprotumumab, a novel monoclonal inhibitor of the insulin-like growth factor-1 receptor (IGF-1R), has recently been approved in the United States for the treatment of thyroid eye disease (TED). In phases 2, 3, and 4 randomized double-masked clinical trials (RCTs),^1,2^ teprotumumab was more effective than placebo in reducing proptosis, diplopia, and inflammation in patients with active TED.^1,3^ Since its introduction, there have been a growing number of studies that have reported on hearing dysfunction after treatment with teprotumumab.

In the phases 2/3 RCTs, hearing impairment adverse events were reported in 10% of patients, with the majority being reversible.^1,2^ In a recent case series of 27 patients, 82% treated with teprotumumab had otological symptoms (tinnitus, ear plugging/fullness, and autophony). In that report, patients were specifically asked (solicited) about otological/hearing symptoms.^4^ Safety studies in general have shown that adverse events are several fold higher when solicited than when using spontaneous reporting, furthermore, spontaneous reporting has been shown to provide larger discrimination in drug–placebo ratio differences and may provide better scrutiny of event relatedness.^5^

Furthermore, only 6 of 27 (22%) patients had baseline and follow-up objective audiometry testing. Given the increasing concern regarding the potential for otological symptoms and hearing dysfunction associated with teprotumumab and the lack of well-controlled hearing assessments published to date, we report the results of a prospective study that investigates both objective (pure tone audiometry) and unsolicited subjective otological symptoms in patients before and after treatment with teprotumumab, with a 6-month follow-up.

This prospective observational study was performed between March 2020 and August 2022, adhering to the tenets of the Declaration of Helsinki, was performed per the Health Insurance Portability and Accountability Act, and was approved by the WCG-IRB (WCG, Puyallup, WA, USA) institutional review board (IRB No. 20210376). All patients who presented to our institution for the treatment of symptomatic TED were considered for study eligibility. All patients provided written informed consent to participate in the study.

Consecutive TED patients who had received all 8 infusions of teprotumumab with a ≥6-month follow-up were included. Patients who were on any other medical therapy for TED or had received rituximab or tocilizumab in the past were excluded. Furthermore, patients who were currently on ototoxic medications were also excluded. Patients received infusions of teprotumumab (10 mg/kg for the first infusion and 20 mg/kg for subsequent infusions) every 3 weeks over 24 weeks to complete 8 infusions.

The primary outcome was change in audiometry. Audiometry testing was completed using a Shoebox Standard audiometer (Shoebox Audiometry Ltd., Ottawa, Canada) at thresholds of 0.25, 0.5, 1, 2, 4, and 8 kHz. This U.S. Occupational Safety and Health Administration-approved device allows assessment of air conduction with masking and software that addresses background noise and unreliable responses. Audiometry testing was completed by a trained technician at baseline (before first infusion on the same day), and before every other infusion. The Shoebox device was calibrated on a regular basis.

Further testing was done at 3 and 6 months after completion of therapy. Criteria for diagnosis of ototoxicity and severity are provided in Supplementary Data. All audiograms were reviewed by an independent audiologist. The mean of thresholds at each frequency per visit, per ear, was calculated to allow comparison between the pretreatment and post-treatment groups.

At each visit, patients were asked whether they had “any changes to their health” before their next infusion and after the last infusion. The same question was asked at the 3- and 6-month follow-up visits. This open-ended question avoided prompting and allowed reduction of potential bias. Change in the activity of TED was assessed using the 7-point clinical activity score (CAS). Earlier case series have shown that hearing dysfunction may occur asymmetrically. Therefore, both ears from each patient were included for analysis. Categorical variables were compared using Pearson χ^2^ or Fisher exact tests, and continuous variables were compared using Student's *t* test. A *p*-value <0.05 was considered statistically significant. All statistical analyses were conducted using SPSS version 22.0 (SPSS, Inc., Chicago, IL, USA).

Fifty-two patients (43 females and 9 males) met the inclusion criteria (Fig. 1). The mean (standard deviation [SD]) age was 49 years (13). The mean (SD) duration of TED before treatment was 32 months (37). All patients were euthyroid and none of the patients were current smokers. All patients completed the full course of treatment. The mean (SD) CAS was 2.4 (1.4) OD (right eye) and 2.2 (1.4) OS (left eye) at baseline. After treatment, the mean (SD) CAS was 1 (1) OU (both eyes) (*p* < 0.05).

**FIG. 1.**
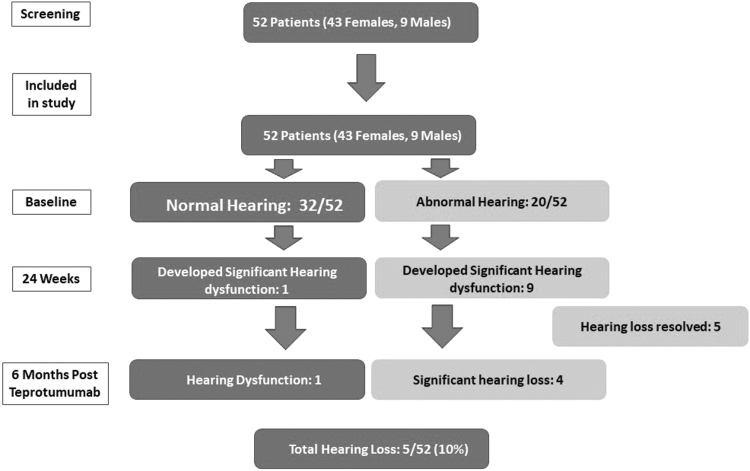
Participant flow diagram and summary of audiometry outcomes. Patients who had hearing thresholds >25 dBHL (decibel hearing level) on any frequency on audiometry were considered to have hearing loss.

Thirty-two of 52 (62%) patients (5 males and 27 females) had normal baseline audiometry. The mean (SD) age of this group was 43 (11) years, while the mean (SD) CAS at baseline was 2 (1.2). After treatment with teprotumumab, 1 patient (3%) had hearing decline on audiometry that had not resolved at 6 months follow-up. This patient's hearing loss was mild (Grade 1) and occurred in one ear. In this group, the mean (SD) hearing threshold (decibel hearing level [dBHL]) was 11.7 dBHL (1.9) at baseline and 12.5 dBHL (2.5) at 6 months follow-up (*p* < 0.05) (normal is <25 dBHL), with a mean decrease of 0.78 dBHL after treatment [confidence interval (CI) −1.3 to −0.3] (Fig. 2A).

**FIG. 2.**
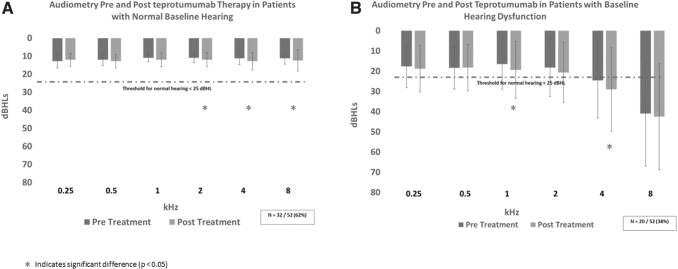
(**A**) Average audiometry at 0.25, 0.5, 1, 2, 4, and 8 kHz, pre- and post-teprotumumab therapy in patients who had normal audiometry at baseline. Dashed line shows the 25 dBHL threshold, beyond which hearing is abnormal. (**B**) Average audiometry pre- and post-teprotumumab therapy in patients who had abnormal audiometry at baseline. Dashed line indicates the 25 dBHL threshold, beyond which hearing is abnormal.

Twenty (38%) of 52 patients (4 males and 16 females) had a baseline hearing dysfunction at 1 or more frequencies. The mean (SD) age of this group was 57 years (12) years and the mean (SD) CAS at baseline was 2.8 (1.4). After treatment with teprotumumab, 10 of 20 patients developed a significant decrease in their hearing on audiometry testing. This decrease in hearing was graded as mild (Grade 1).

The mean (SD) age of this group was 63 (11) years, while the mean (SD) age of those who did not have a hearing change was 52 (9) years (*p* < 0.05). At 6 months follow-up, 6 of 10 patients had complete resolution of hearing changes (Fig. 1). All 4 patients who did not have resolution had severe hearing loss at baseline. At baseline, the mean (SD) threshold (dBHL) in this group was 23 dBHL (13). At 6 months after the last infusion, the mean (SD) threshold was 25 dBHL (15) (*p* < 0.05) (normal is <25 dBHL), with a mean decrease of 2 dBHL after treatment [CI −3.4 to −0.6] (Fig. 2B).

Patients with abnormal baseline audiometry were older (*p* < 0.05) and had a higher CAS than those with normal baseline audiometry (*p* < 0.05). Only 1 of 32 patients (3%) with normal baseline hearing had new onset hearing loss (on audiometry) at 6 month follow-up. This patient's hearing loss was mild (Grade 1) and the patient was asymptomatic during treatment and at 6 months follow-up. Four out of 20 (20%) patients who had baseline hearing dysfunction had worsening of their hearing after teprotumumab therapy. All hearing loss in this group was unilateral and mild (Grade 1). Combining the 2 groups of patients, a total of 5 of 52 (10%) patients had a significant but mild (Grade 1) hearing loss after teprotumumab therapy at 6 months follow-up.

At baseline, 6 of 52 (12%) patients reported otological symptoms. Two patients reported tinnitus, while 3 reported bilateral hearing decline and 1 had bilateral hyperacusis. After initiation of treatment, 15 (29%) patients reported new otological symptoms that had not resolved by the end of therapy (week 24). Of these, 4 of 15 patients had plugged ears, 5 of 15 patients had hearing changes, and 4 of 15 patients had the sensation of fullness in their ears, while 3 of 15 patients had tinnitus (1 patient had tinnitus and hearing changes together). At 6 months follow-up, 11 of 15 patients had complete resolution of symptoms. The mean (SD) age of those who reported otological symptoms at baseline was 60 (10) years, and 48 (13) years in those who had no symptoms at baseline (*p* < 0.05).

In our study, in those with normal baseline audiometry, hearing loss was rare, with only 1 of 32 patients being affected at 6 months follow-up. This patient remained asymptomatic from baseline to 6 months follow-up. Overall, in this group, there was a mean decrease of 0.78 dBHL after treatment [CI −1.3 to −0.3]. To put this in context, the sound of normal breathing is as loud as 10 dBHL.^6^

Furthermore, over one-third of patients had hearing dysfunction on audiometry at baseline. This group was significantly older and had a higher CAS than the population who had normal hearing at baseline. In the United States, the prevalence of hearing-related issues in this age group ranges from 19% to 29% (19% prevalence in ages 45–64 years and 29% for those aged >65 years).^7^

The increased incidence of hearing dysfunction in TED patients at baseline might be explained by the potential impact of autoimmune thyroid disease and/or its treatment on hearing. Of those with an abnormal audiogram at baseline, 10 of 20 patients had hearing loss at 24 weeks, with 6 of 10 patients (60%) improving by the 6-month follow-up visit. Four of the 10 patients (40%) did not improve. Our results support previous findings that suggest that baseline hearing loss was a significant risk factor for further hearing loss after teprotumumab therapy.^4^

In this study, 29% reported new subjective otological symptoms, this differs from the findings of the phases 2 and 3 RCTs with teprotumumab where only 10% spontaneously reported hearing-related symptoms, with 5% of these judged to be drug related.^1,2^ Furthermore, like the RCTs, the majority (11 of 15 patients) had complete resolution of their symptoms. These findings reinforce the importance of quantifiable baseline audiometry to monitor changes in hearing related to therapy.

The findings of our study may be explained by previous study that suggests that the IGF-1R is expressed in the developing inner ear where it maintains and prevents apoptosis of hair cells through downstream signaling pathways in the cochlea.^8^ It is also thought to promote the regeneration of cochlear ribbon synapsis, and it is involved in the synaptic neurotransmission of the cochlear nuclei.^8^ A definitive causal relationship has not been found between IGF-1R inhibition and hearing changes. Our study was limited by a follow-up of 6 months.

We noted a gradual improvement in the audiometry and symptoms of those with hearing loss/otological symptoms with time. It is possible that with longer follow-up, the prevalence of these signs and symptoms may have reduced. In a recent study, 11 of 22 patients treated with teprotumumab had hearing loss. This study differed from ours in that it had fewer patients and post-treatment follow-up varied between 0 and 496 days, whereas our follow-up was longer and the same for all patients.^9^

We used the Shoebox digital audiometer as opposed to conventional audiometry. However, previous study has shown that the Shoebox audiometer was deemed clinically accurate and had results within five dBHL for both air and bone conduction.^10^ In 1 study of 49 patients, Shoebox audiometry demonstrated a sensitivity between 0.87 and 1 and specificity between 0.9 and 0.95 for frequencies ranging from 0.5 to 4 kHz in identifying patients with hearing loss diagnosed by conventional audiometry.^11^

Comprehensive testing for ototoxicity typically involves testing with bone conduction and testing for word recognition, along with collection of information on potentially ototoxic conditions, for example, diabetes and hypertension. We were unable to include these in our study and envisage that this study will stimulate further work. Finally, we did not specifically ask patients about hearing disturbance, but asked patients whether they had “any changes to health,” this may make it less likely that patients reported hearing symptoms.

Long-term hearing loss in TED patients with normal baseline hearing is rare after treatment with teprotumumab (incidence of 3%). In those who already had hearing dysfunction before therapy, worsening was seen in 20% (4 of 20) patients. Patients with baseline hearing dysfunction are at significantly greater risk of hearing loss and would benefit from the institution of a screening program. We invite further research to help address appropriate adjustment in treatment in patients with hearing loss.

## Supplementary Material

Supplemental data
