# Supplementary material for: A Prospective Study Examining Audiometry Outcomes Following Teprotumumab Treatment for Thyroid Eye Disease
Source: Thyroid. 2024 Jan 16;34(1):134–7. doi: 10.1089/thy.2023.0466 (PMC10818043; doi:10.1089/thy.2023.0466)
Supplement: Supplemental data [file Suppl_Data.docx]

# Supplement 1 – Defining Significant Change on Audiometry and Grading Severity

Significant change on audiometry was defined by using the American Speech-Language-Hearing Association (ASHA) criteria for ototoxicity (>20 dB pure-tone threshold shift at one frequency, >10 dB shift at each of 2 consecutive frequencies, or threshold response shifting to “no response” at 3 consecutive test frequencies).^1^ Thresholds below 25 dBHLs were considered normal for each frequency, as per the manufacturers guidelines and the National Institute of Deafness and other Communication Disorders’ (NIDCD) guidelines^2^.

The severity of significant changes on audiology was graded according to the Common Terminology Criteria for Adverse Events (CTCAE) ototoxicity scale^3^. Severity is graded according to threshold changes: grade 1 = mild impairment (increase of 15-20 dBHLs at 2 contiguous frequencies), grade 2 = moderate (≥25 dBHLs at 2 contiguous frequencies), grade 3 = severe (≥25 dBHLs at 3 contiguous frequencies) and grade 4 = profound loss (>80 dBHLs at 2kHz or above).

**References**

1. Rizk, HG, Lee, JA, Liu, YF, et al. Drug-Induced Ototoxicity: A Comprehensive Review and Reference Guide. Pharmacother J Hum Pharmacol Drug Ther 2020;40(12):1265–1275; doi: 10.1002/PHAR.2478.

2. Anonymous. What the Numbers Mean: An Epidemiological Perspective on Hearing | NIDCD. n.d. Available from: https://www.nidcd.nih.gov/health/statistics/what-numbers-mean-epidemiological-perspective-hearing [Last accessed: 10/6/2022].

3. Cancer Institute N. Common Terminology Criteria for Adverse Events (CTCAE) Version 4.0. 2009.
